# Supplementary material for: Efficient Ocular Delivery of VCP siRNA via Reverse Magnetofection in RHO P23H Rodent Retina Explants
Source: Pharmaceutics. 2021 Feb 6;13(2):225. doi: 10.3390/pharmaceutics13020225 (PMC7914601; doi:10.3390/pharmaceutics13020225)
Supplement: Supplementary file 1 [file pharmaceutics-13-00225-s001.pdf]

*pharmaceutics*

MDPI

# Supplementary Materials: Efficient Ocular Delivery of VCP siRNA via Reverse Magnetofection in RHO P23H Rodent Retina Explants

**Merve Sen, Marco Bassetto, Florent Poulhes, Olivier Zelphati, Marius Ueffing and Blanca Arango-Gonzalez**

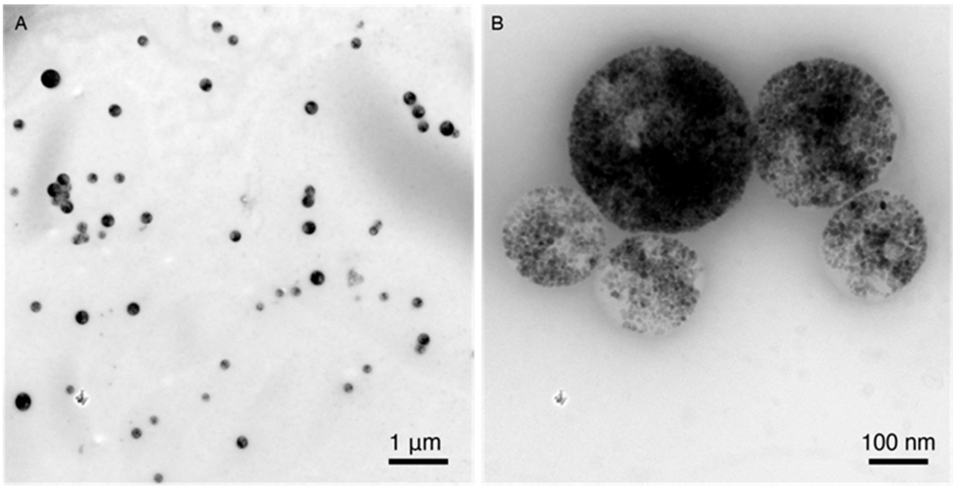


**Figure S1.** TEM images of XPMag. (**A**) XPMag is composed of spherical particles and (**B**) has a diameter smaller than 200 nm that contains smaller iron oxide particles of 5nm. Scale bars 1µm and 100 nm, respectively.

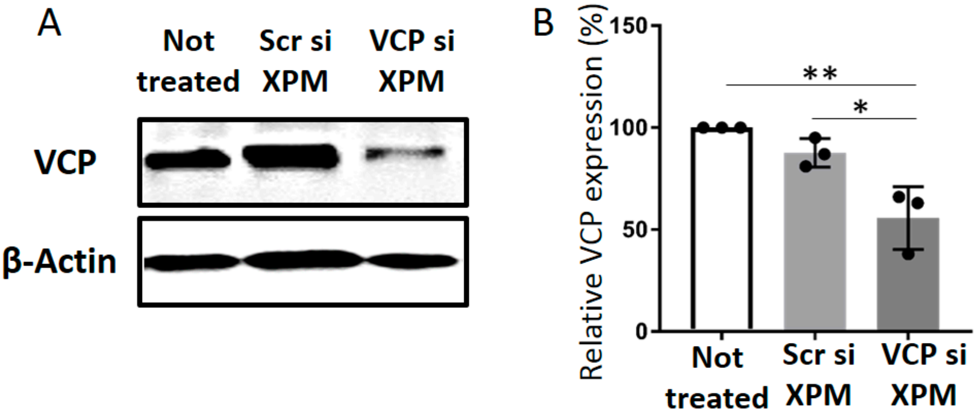


**Figure S2.** XPMag enhances efficient in vitro VCP silencing in human hTERT-RPE1 cells via classical Magnetofection. (**A**) Representative images of Western blotting for the cell lysates of untreated, 50 nM scrambled siRNA/XPMag-treated, and 50 nM VCP siRNA/XPMag-treated human hTERT-RPE1 cells using beta-actin as a loading control. (**B**) VCP bands (97 kDA) intensities were normalized to the corresponding band of the loading control beta-actin (42 kDa), and results were expressed as relative VCP expression for 50 nM VCP siRNA (%). Data are presented as means ± SD, and one-way ANOVA analysis was performed at * $p < 0.05$ and ** $p < 0.01$. Dark circles represent each replicates, $n = 3$. Abbreviations: VCP si: VCP siRNA, Scr si: Scrambled siRNA, XPM: XPMag, kDa: Kilo Dalton.

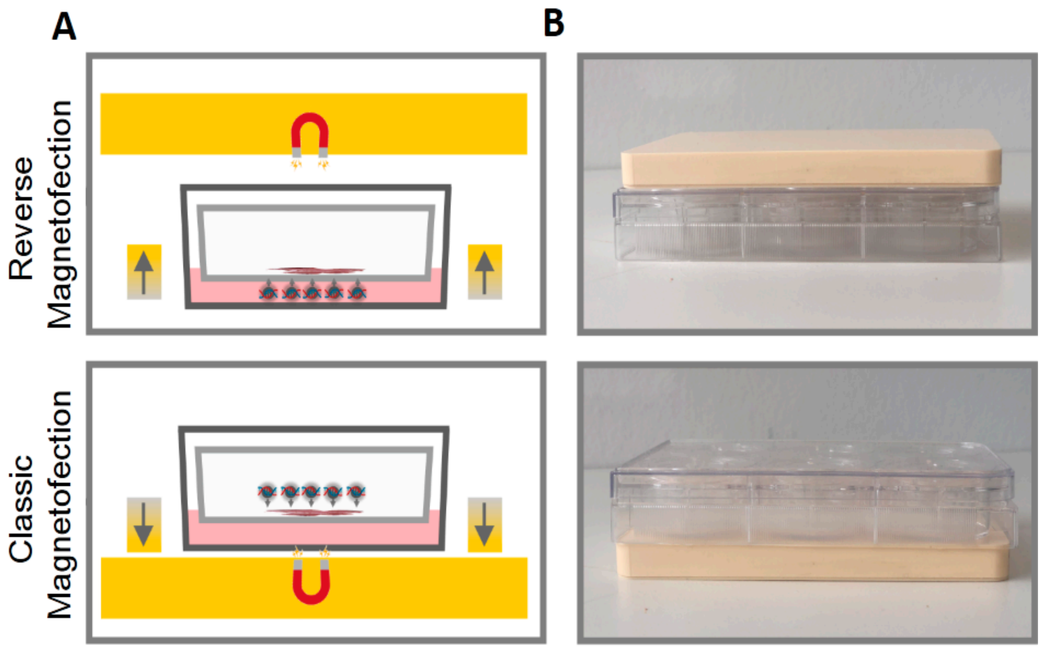


**Figure S3.** Schematic (**A**) and photographic (**B**) representations of Reverse and classical Magnetofection techniques used for transfection of siRNA in organotypic retinal cultures. In contrast to Reverse Magnetofection, classical Magnetofection is achieved by adding siRNA/XPMag MNPs onto the retinal explant, followed by placing the super-magnet under the culture plate to attract the complexes from the GCL to the RPE.

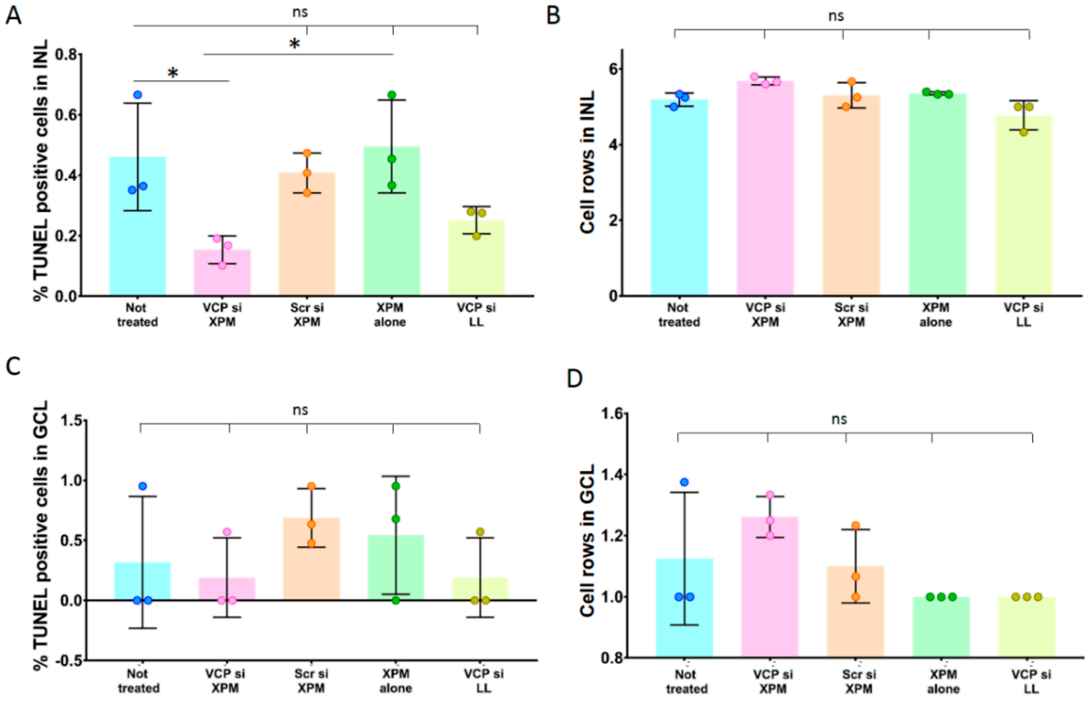


**Figure S4.** Reverse Magnetofection does not induce cellular toxicity in the inner retina in RHO P23H retinal explants. Retinae of RHO P23H transgenic rats were explanted at postnatal day 12 and cultured for three days. Retinae were treated with 50 nM VCP si/XPMag, 50 nM Scr si/XPMag, and XPMag alone by Reverse Magnetofection and VCP si/Lullaby by lipofection. (**A**) Bar graph shows the percentage of TUNEL-positive cells in the INL. After treatment with VCP siRNA/XPMag via Reverse Magnetofection, a significant decrease in the percentage of cell death was observed compared to the other groups. (**B**) Quantification of the cell rows in INL. (**C**) Bar graph shows the percentage of TUNEL-positive cells in the GCL. After treatment with VCP siRNA/XPMag via Reverse Magnetofection, there was no further cellular toxicity. (**D**) Quantification of the cell rows in GCL.

The values were obtained by scoring several images (circles) from 3 retinae ($n = 3$) per treatment for: Not treated, VCP si/XPM, Scr si/XPM, XPM alone, and VCP si/LL. Data plotted as mean ± SD. One-way ANOVA, * $p < 0.05$. Abbreviations: VCP si: VCP siRNA, Scr si: scrambled siRNA, XPM: XPMag, LL: Lullaby, FL: fluorescence, INL: inner nuclear layer, and GCL: ganglion cell layer.

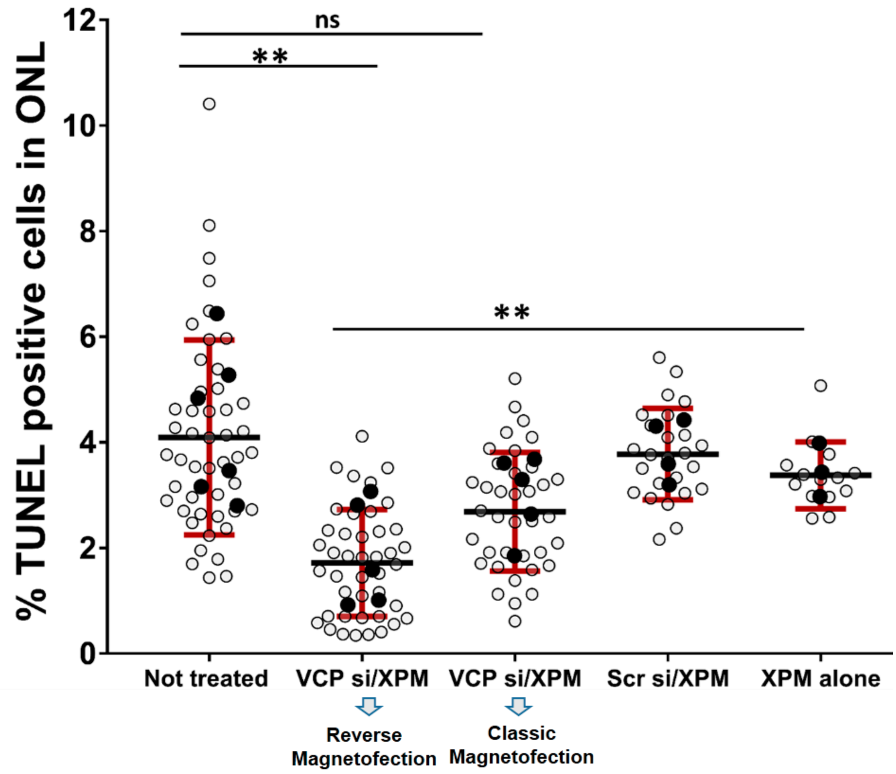


**Figure S5.** Cell death analysis for different Magnetofection techniques in RHO P23H retinal explants. The VCP siRNA/XPMag complexes in RHO P23H organotypic retinal culture were either added into the culture medium (Reverse Magnetofection, Figure S1A,B) or onto the retina (classic Magnetofection, Figure S1A,B). Retinae of RHO P23H transgenic rats were explanted on postnatal day 12, cultured *in vitro* for three days. RHO P23H retinae were treated with 50 nM VCP si/XPMag, 50 nM Scr si/XPMag, and XPMag alone by Reverse Magnetofection and 50 nM VCP si/XPMag by classic Magnetofection. Results were calculated as the number of TUNEL positive nuclei of ONL normalized to the total number of nuclei in the ONL and were expressed as the percentage of TUNEL positive cells. Values were quantified by scoring several images (open gray circles) from at least three retinae (black closed circles) per treatment. Data are presented as means ± SD, and one-way ANOVA analysis was performed at ** $p < 0.01$. Abbreviations: VCP si: VCP siRNA, Scr si: scrambled siRNA, XPM: XPMag, ns: non-significant.

**Table S1.** List of antibodies used in this study.

| Antigen | Source | Cat. Number | Dilution IF/IHC | Dilution WB |
|---|---|---|---|---|
| VCP | Thermo Fischer Scientific | MA3-004 | 1:200 | 1:1000 |
| IgG Alexa Fluor™ 568 dye-conjugated goat anti-mouse IgG | Molecular Probes | A 11031 | 1:500 | |
| Alexa Fluor™ 488 dye-conjugated goat anti-rabbit IgG | Cell Signaling Technology | A 11034 | 1:500 | |
| Rhodopsin | Merck Millipore | MAB5356 | 1:300 | |
| Iba1 | Fujifilm Wako Chemicals | 019-19741 | 1:200 | |
| GFAP | Merck Millipore | G3893 | 1:500 | |
| β-Actin | Cell Signaling Technology | 4970 | | 1:1000 |
| Anti-mouse IgG kappa binding protein-coupled to horseradish peroxidase | Cell Signaling Technology | 7076 | | 1:2000 |
